# Households Contrasted

**Published:** 1876-03

**Authors:** 


### HOUSEHOLDS CONTRASTED.

A GORGED anaconda is the mildest animal in nature. You may kick him until you are tired, and he won't take the trouble to raise his head or give a hiss. We have remembrances as fresh as of yesterday, of going out to the pig-pen of a frosty fall morning, and witnessing the changing mood of the occupants while waiting for the men to throw in the corn. How they would squeal, and grunt, and bite, and snap at each other! these modes of expression becoming "excelsior" in geometrical progression with each passing minute of delay. With the first mouthful, "order reigns in Warsaw." A few minutes later, a wag of delight takes hold of the tail, and at the "closing stretch," pig spoons to pig, and side by side they while their time away in delicious, dozy grunts. Pigs and people are not far apart. There are men, and—shall we say it?—women, too, who, before they get their breakfast in the morning, are as ferocious as she-tigers. They have brutalized themselves by late dinners, or over-hearty suppers the preceding evening, or they have sat up until midnight, or later, in doing what they considered indispensable—working, it may be in actual weariness and suffering, with the effect, that when the morning comes, they are not rested, not refreshed, and, as a consequence, the nervous system is deranged; and if they are up soon enough for family worship, they come into the room where glad children and servants (for both children and servants being healthy, are light-hearted in the mornings) are all in waiting, with a tremendous scowl on the countenance, or a sheepish, slovenly indifference, according to the temperament of the human animal. But not the honey droppings of the "Word;" not the glad songs of praise from other lips; or thanks for the nightly deliverances or the morning mercies, the bright sunshine, the dancing fire on the hearth, while the frost is at the window; not the consideration that every child is the picture of health, and a well-spread board, white and clean and smoking and abundant, is ready, without even the trouble of ordering; not all these considerations are sufficient to mellow down that ugly nature, but it drawls along to the table to whine and complain and growl and fret. If a child happens to laugh outright in its gladness, there is an instantaneous snap at it, if, indeed, it is not driven from the table, or slapped over with the hand. If the toast is not browned to a turn, or the steak is the tiniest bit over or under-done, the cook is raved at with the savageness of a polar bear. If the table-maid happens in her haste to trip a toe or let fall a roll, noise enough is made about it to fill a barn; and if a child overturns a cup, a hyena comes up in the "dissolving view."

Reader! what think you? does this picture of a daily reality in more households than a dozen, suit you in whole, or even small part? Be counseled in time, that one of two results will fall to your lot: if, in humility and repentance towards God and man, you do not promptly seek a remedy against so low a crime, you will either die prematurely, or end your days in an asylum, if not in a worse place still. If you begin a day in so ungodly a manner, it is a "bad beginning" for you; the whole of that day will be a cloud; the night will set in with humiliating or fierce remorses, and it will be a day lost—lost forever. If it were only lost to you, it is of comparatively small moment, except to yourself. But that is not all. You clouded over a sunshine which God had sent to gladden the members of your family. You robbed them of God's good gift, there will be blasting in the repetition, blasting to the body, and curses to the soul, ending never! "That's true, Doctor; every word of it is as true as gospel, what you say about parents being pleasant in families. I've seen it in more cases than one or two." So said neighbor P., who lives a block or two away, in Seventeenth street. He ran on so glibly in this style, ever so long, and we couldn't stop him; so we let him run! What a grand good plan it is, in another direction, to stop a verbosity, as Rev. Dr. C. would say, just to say nothing at all. Why, the longest tongue in nature will stop short in "two forty," if you only don't say a single thing. It's because we have tried it that we recommend it so confidently; and if you have any sly malice to gratify, you can indulge it so effectually and in so quiet a way, too! The more you don't say any thing, the longer will the remorse of meanness burn with unquenching fierceness afterwards. But to friend P.'s speech. "You know, Doctor, I have one of the finest wives in the world, so I am not personal. 'O Mrs. P.!' said two dear young girls, 'if mother would only be as lively and kind-tempered as you are! You seem to be always cheerful, and in such good spirits! but mother is always cross.' " On inquiry, we learned that a merchant of great wealth had died before his time in Brooklyn, and left his widow with three children. Her temper was perfectly devilish. It shortened his days. While in the hot pursuit of business, his mind was diverted; but when his fortune was made, and he had leisure to be at home, and to enjoy his children, the curse of a shrew came upon him, and he had not the courage or the moral power to apply a remedy. The result was, that in this case, the young son left the house — had not slept in it for three years. He found greater pleasure in running after the "machine," and slept in the engine-house. The presumption of charity is, that the woman was deranged.

But a merited tribute to our neighbor. He has reared a family of sons and daughters in New-York City. Model children are they all. The sons are handsome, manly, and of moral character without the film of a blot. The daughters, single and married, are affectionate as children, and notable in the relations of wife and mother. The father and mother have grown old in lovingness within and charity without. The frequent reunions of parents and children and grandchildren, are tableaux of earthly delight; and with peace and plenty, and blessed unity combined, they bid fair to end their days in gladness and sunshine, and all largely owing to a cheerful conduct in the household; showing, by the way, that a family of children can grow up in a city, healthy in body, blameless in morals, and unchallenged in business integrity, by making home attractive.

